# Atmospheric Pressure Chemical Ionization Gas Chromatography Mass Spectrometry for the Analysis of Selected Emerging Brominated Flame Retardants in Foods

**DOI:** 10.1038/srep43998

**Published:** 2017-03-10

**Authors:** Surong Lv, Yumin Niu, Jing Zhang, Bing Shao, Zhenxia Du

**Affiliations:** 1 Beijing Advanced Innovation Center for Food Nutrition and Human Health, China Agricultural University, Beijing 100193, China; 2 College of Science, Beijing University of Chemical Technology, Beijing, 100029, China; 3 Beijing Key Laboratory of Diagnostic and Traceability Technologies for Food Poisoning, Beijing Center for Disease Prevention and Control, 100013, China

## Abstract

Emerging brominated flame retardants (eBFRs) other than polybrominated diphenyl ethers (PBDEs), polybrominated biphenyls (PBBs) and their derivatives in foods have been in focus in recent years due to their increasing production volumes, indefinite information on toxicities and the lack of data on occurrence in environments, foods as well as humans. In this study, gas chromatography was coupled to an atmospheric pressure chemical ionization-tandem mass spectrometry (APGC-MS/MS) for the analysis of six eBFRs in pork, chicken, egg, milk and fish. A short section of unpacked capillary column coupled to the end of the analytical column was applied to improve the chromatographic behaviors of high boiling point compounds. The method was comprehensively validated with method limit of quantification (mLOQ) lower than 8 pg/g wet weight (w.w.). Samples from Chinese Total Diet study were quantified following the validated APGC-MS/MS method. 2,3,4,5-pentabromo-6-ethylbenzene (PBEB), hexabromobenzene (HBB), pentabromotoluene (PBT) and 1,2-bis(2,4,6-tribromophenoxy)ethane (BTBPE) were most frequently detected in samples. The highest concentration was found in fish with 351.9 pg/g w.w. of PBT. This is the first report on the presence of PBT in food samples with non-ignorable concentrations and detection rate.

In the past few decades, flame retardants (FRs) have been widely used in a variety of products, such as plastics, electronic equipments, furnitures and textiles to reduce their flammability^1^. During the use and disposal of corresponding products, FRs may be released into the environment^2^,^3^. Considering their adverse effects on both humans and the eco-environment, worldwide strict bans have been imposed on the use of formulations containing penta- and octa-brominated diphenyl ether^4^. Therefore, some emerging brominated flame retardants (eBFRs), such as HBB, PBT, BTBPE, 2,3,5,6-tetrabromo-p-xylene (pTBX), and hexachlorocyclopentadienyl-dibromocyclooctane (DBHCTD) have been increasingly manufactured as replacements^5^,^6^. In recent years, these eBFRs have been manufactured in China^5^,^7^,^8^ and found in different environmental matrice^8^,^9^. Some of these contaminants tend to bioaccumulate in animals and induce oxidative stress damage^10^,^11^. They are also known to transfer to foods through different pathways^12^,^13^. Food is one of the major routes of FR human exposure together with inhalation of indoor air and dust. However, there is little information on the occurrence of these eBFRs in food. In 2009 European Food Safety Authority (EFSA) called for data on the occurrence of BFRs including eBFRs in foods. In the 215 analytical results corresponding to HBB, all values were < LOQ (0.01 μg/kg whole weight). For the 215 analytical results corresponding to BTBPE, only 11% data were above LOQ (0.01 μg/kg whole weight)^14^. Therefore, EFSA asked its Member States to monitor the presence of eBFRs in food over the next two years to gather more data for risk assessment^15^.

Conventionally, gas chromatography coupled to mass spectrometry (GC-MS) using negative chemical ionization (NCI) source has been widely applied in the field of BFRs analysis to obtain higher sensitivity^6^,^11^,^16^. However, this technique has lower selectivity and specificity for eBFRs since only two isotopes of bromine atom (*m/z* 79 and 81) can be monitored. There is a likelihood that only bromine ion isotopes can be monitored to allow discrimination between co-eluting brominated compounds, which can lead to cross-talk of bromine-containing compounds. GC-MS operating in electron ionization (EI) mode can obtain significant fragmentations, and therefore, better selectivity than NCI. However, as a hard ionization mode, it still cannot obtain higher sensitivities for molecular ions^17^,^18^. New attempts using liquid chromatography coupled to orbitrap mass spectrometry (LC-MS/MS) using atmospheric pressure photoionization (APPI) mode have been reported to detect some selected eBFRs (PBEB, HBB, BTBPE and DBHCTD) in fish with instrument limit of quantification (iLOQ) in the range of 0.5–59 pg on-column levels^19^,^20^. However, displacement reactions were the most dominant pathways in APPI mode, the main precursor ions were observed as unstable [M-Br + O]^−^ or [M + O_2_]^−^ other than molecular ion [M-H]^−^. Recently, APGC-MS/MS with advantage over GC-EI-MS and GC-NCI-MS for the generation of predominant diagnostic molecular ions and characteristic fragment ions has been introduced to analyze polybrominated diphenyl ether (PBDEs) in cream at pg/g levels^21^. With the strict bans on the using of PBDEs and polybrominated biphenyls (PBBs) and the increasing volumes of eBFRs, more concerns have been focused on these emerging compounds since the limited experimental data indicated their bioaccumulation and persistence^15^,^22^. Therefore, more sensitive analytical method for the determination of eBFRs was necessary to assess the potential human risk by dietary intakes.

The main goal of this study was to establish a more sensitive method based on APGC-MS/MS for the analysis of eBFRs in main foods of animal origin such as pork, chicken, egg, milk and fish. The Chinese Total Dietary Study (TDS) samples were collected for the monitoring occurrence of six selected eBFRs.

## Results and Discussion

### Optimization of Mass Spectrometric Parameters

The chromatographic retention time, precursor and product ion transitions and MS conditions for analysis of eBFRs using GC-APCI-MS/MS, GC-EI-MS/MS and GC-NCI-MS are listed in Table 1. When using GC-NCI-MS, only two isotopic peaks of bromine atom (*m/z* 79 and 81) are dominant in the mass spectrums of emerging brominated compounds (Table 1), which are in line with previous reports^6^,^11^,^23^,^24^.

The GC-EI-MS mass spectrums of p-TBX, PBEB, HBB and PBT show apparent molecular ions as well as a series of fragment ions associated with loss of bromine atoms, the molecular ions M^+·^ are selected as precursor ions for GC-EI-MS/MS analysis. BTBPE presents a base peak at *m/z* 356.5, corresponding to loss of the [C_6_H_2_OBr_3_]. And the most intense peak of DBHCTD was *m/z* 107.1, corresponding to loss of the [C_10_H_12_Cl_4_Br_2_], resulting in low response for the molecular ion. Therefore, [M-C_6_H_2_OBr_3_] and [M-C_10_H_12_Cl_4_Br_2_] are selected as precursor ions for BTBPE and DBHCTD analysis, respectively. The specific EI-MS/MS experimental conditions of eBFRs are displayed in Table 1.

It is known that, using modifiers such as water^25^, methanol^26^ or 1% formic acid^21^ solution, in the ion source chamber, promotes the generation of protonated molecular ion species. Thus, two different ionization mode, charge transfer and proton transfer, were also compared to study the impact of modifiers and achieve the most abundance of molecular ions. The results indicated that the eBFRs favored to ionization in positive mode and scarcely ionized in negative. For all the eBFRs studied in this work, unlike the behaviors of PBDEs on APGC-MS/MS^21^, the intensities of charge transfer ions M^+·^ are the most abundance rather than proton transfer ions [M + H]^+^ (Supplementary Fig. S1). Compared the structure of PBDEs and eBFRs, it is clear that the existence of oxygen atom in PBDEs can provide lone pair electrons and proton transfer ions are tend to produced. Furthermore, the presence of modifiers leads to remarkable decreasing of the M^+·^ intensity. Thus, the whole experiment process was conducted under “dry” condition (without adding modifiers). After the optimization of the ionization mode, the corona current, cone voltage and collision energy were optimized to maximize the abundance of the precursor ions and product ions. The results of optimization are also detailed in Table 1.

**Figure 1:**
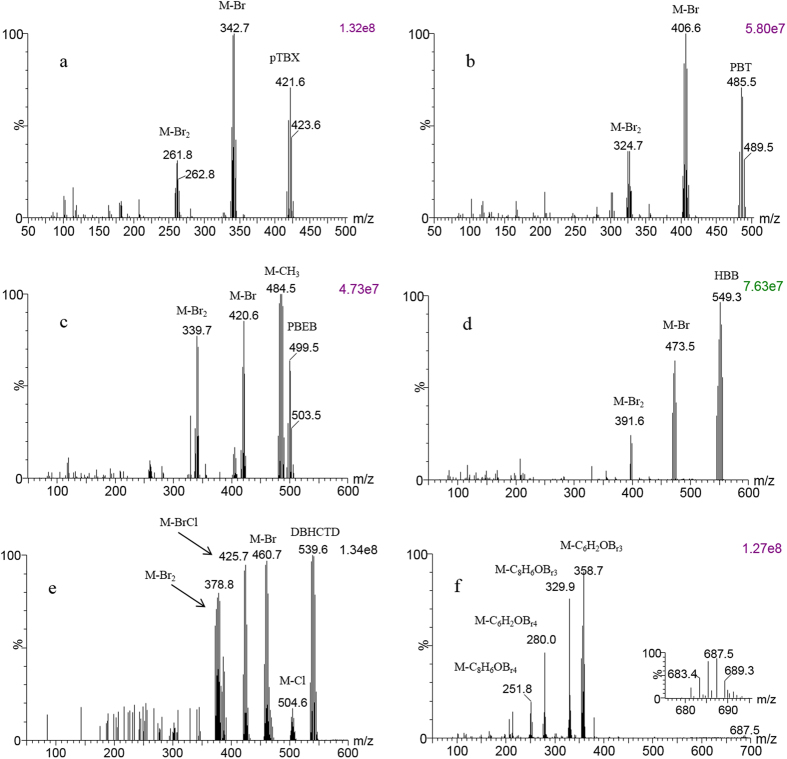
The APGC-MS spectrums of EBFRs eBFRs (**a**-pTBX; **b**-PBT; **c**-PBEB; **d**-HBB; **e**-DBHCTD; **f**-BTBPE) after in-source collision-induced dissociation under full scan mode. The cone energy was 35 V in Fig. 1a–d and 40 V in Fig. 1e,f, 30 V in Fig. 1e and 25 V in Fig. 1f.

Fragmentation of the six analytes in in-source collision-induced dissociation (CID) mode (Fig. 1) was studied. The molecular ions M^+·^ were selected as precursors to generate product ions at different collision energies in the range of 5–50 eV. The optimal results are listed in Table 1. PBT, pTBX and HBB, which structure based on benzene ring substitute by bromine atom, generated the losses of one or two bromine atoms. PBEB lost methyl firstly because of branched carbon chain. The *m/z* 142.7 of DBHCTD corresponding to the loss of [C_10_H_12_Cl_3_Br_2_], while the *m/z* 105.1 corresponding to the further loss of chlorine atom. The *m/z* 358.7 of BTBPE corresponding to the loss of [C_6_H_2_OBr_3_], while the *m/z* 279.8 corresponding to the further loss of bromine atom. The most intense transitions were used for quantification, while other transitions were used for qualification.

### Optimization of Chromatographic Conditions

Injector temperature is a crucial parameter for polybrominated compounds. A previous study showed that about 1~3% of DBHCTD was degraded by debromination reaction when injector port was maintained at 250 °C^27^. In the current study, degradation was observed (about 6%) when injector temperature increased to 280 °C. However, this was the optimal temperature for the other five analytes (Supplementary Fig. S3). As a compromise, the injector temperature was set at 280 °C.

**Figure 2:**
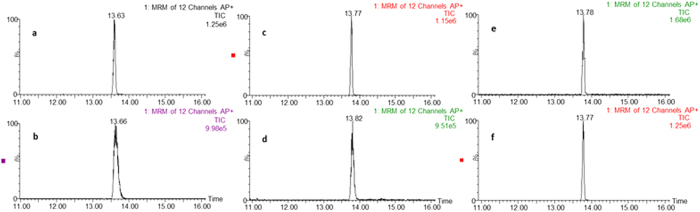
The chromatographams of BTBPE. (**a**) The first injection of 10 μg/L BTBPE standard with transfer line temperature 310 °C, (**b**) 10 μg/L BTBPE standard after 25 repeat injections with transfer line temperature 310 °C, (**c**) the first injection of 10 μg/L BTBPE standard with transfer line temperature 310 °C and a deactived column in transfer line, (**d**) 10 μg/L BTBPE standard after 25 repeat injections with transfer line temperature 310 °C and a deactived column in transfer line. (**e**) The first injection of 10 μg/L BTBPE standard with transfer line temperature 350 °C and a deactived column in transfer line, (**f**) 10 μg/L BTBPE standard after 25 repeat injections with transfer line temperature 350 °C and a deactived column in transfer line.

The APGC-MS/MS technique has been successfully applied to the analysis of pesticides^25^, polycyclic aromatic hydrocarbons^28^, PBDEs^21^ and dioxins^29^ with high sensitivity and excellent chromatographic behavior. However, in this work, chromatographic peak tailing, retention time delay and reduced intensity for high boiling point eBFRs were occurred Fig. 2a,b. This may attribute to the cooling effects of the transfer line, when high boiling point analytes exit the heated transfer line, there areapproximately 2 mm capillary column protruding out of the heated transfer line before entering ion chamber. To overcome the limitation, temperature of heated transfer line was attempted to increase. However, the highest operation temperature of DB-5MS column was 325 °C. Therefore, an unpacked column was added at the end of analysis column and temperature of heated transfer line was optimized between 300 and 380 °C with an interval of 10 °C. As shown in Supplementary Fig. S4, properly rise of the transfer line temperature was beneficial to intensity of analytes except for DBHCTD, while it stared to decrease over 350 °C. As a consequence, the temperature of heated transfer line was hold on 350 °C. From Fig. 2c,d, the peak tailing was still existed for BTBPE after repeated injections with the transfer line temperature 310 °C with unpacked column at the end of the analysis column. However, with the optimized temperature (350 °C), chromatographic peak tailing, retention time delay and reduced intensity for BTBPE were greatly improved (Fig. 2e,f). Therefore, the improvement was due to the increased temperature of transfer line. In addition, the length of unpacked column was optimized at 55 cm (a), 70 cm (b) and 85 cm (c), and the retention time intensity and peak shape were barely changed with a 0.25 min shift of retention time (Supplementary Fig. S5). The influence of the lengths of unpacked column was not significant on both rentention time and intensity. To maintain consistent retention time, 70 cm was selected. This column tandem method can be used for reference of high boiling point compounds using APGC-MS/MS analysis.

### Sample Preparation and Matrix Effects

The sample extraction was performed using accelerated solvent extraction (ASE). The extraction time, pressure and temperature of extraction cell were optimized as *n*-hexane/acetone (3:1, v/v) in 100 °C, 1500 psi and 10 min per static extraction process (Supplementary Fig. S6). After sample extraction, the extracts were further purified using multilayer silica gel packing column with a little modification based on previous article^12^. It is well known that matrix effects, commonly matrix suppressions, are a major complicating factor of analytical methodologies for biological matrices by LC-MS, especially in electrospray ionization (ESI) source. It was widely believed that elaborate sample preparation could reduce the matrix effects. In previous article^20^ for the determination of eBFRs in fish using LC-APPI-MS, although additional gel-permeation chromatography process was added to further sample purification, 40% matrix suppressions were observed. The matrix effects in this study, based on the slope ratios between matrix-matched curves and solvent standard curves were summarized in Supplementary Table S2. The matrix effcts of six analytes as well as internal standards were all evaluated, because of the lacking of cooresponding isotope internal standards of several analytes. When using isotope internal standards of other compounds, it is nessessary to evaluate whether they have similar matrix effect. Isotope interanl standards of other compounds could be used for correction only if the two have similar matrix effect. The results indicate that few matrix suppressions are observed for the slope ratios for most analytes ranging from 0.8 to 1.10, which is considered low matrix effects^30^ and solvent standard curves can be used for quantification.

### Method Characterisation

Linearity was studied by analyzing standard solutions in the following ranges: 0.02–2 pg/μL (PBEB), 0.05–2 pg/μL (pTBX, PBT, HBB, BTBPE) and 0.25–5 pg/μL (DBHCTD). All eBFRs showed a good linearity with r^2^ > 0.99 and residuals lower than 20%. APGC-MS/MS exhibited significant advantage over GC-NCI-MS and GC-EI-MS/MS (Table 2).

In contrast to GC-NCI-MS and GC-EI-MS/MS, the APGC-MS/MS sensitivities based on instrumental limit of quantifications (iLOQ) (signal-to-noise ratio 10) were enhanced 4–40 times, which are lower than 50 fg with 1 μl injection except for DBHCTD. The method limit of quantifications (mLOQs) were ranged from 1–8 pg/g wet weight (w.w.) for APGC analysis, while others, like LC-APPI-MS, were 2–190 pg/g w.w. in fish samples^20^. The mLOQs of some research were described as ng/g lipid weight (l.w.). To compare with others, mLOQ was converted to ng/g l.w. based on the lipid content of different food, and the mLOQ was between 0.007–0.178 ng/g l.w. These values were lower than 0.01–15 ng/g l.w. for PBT and pTBX^12^, 0.49 ng/g l.w. for DBHCTD^8^,0.596 ng/g l.w. for PBEB and 0.444 ng/g l.w. for HBB using GC-NCI-MS^31^. The accuracies were based on the recoveries for all eBFRs in blank fish, egg, chicken and milk samples ranged from 80% to 120%, with the relative standard deviations (RSDs) lower than 19.6% based on the six replicates shown in Table 3.

### Application to the Chinese total dietary samples

The developed method was applied to several foods of animal-origin from the Chinese TDS in 2011 including pork, chicken, egg, milk and fish and the results are summarized in Table 4.

**Figure 3:**
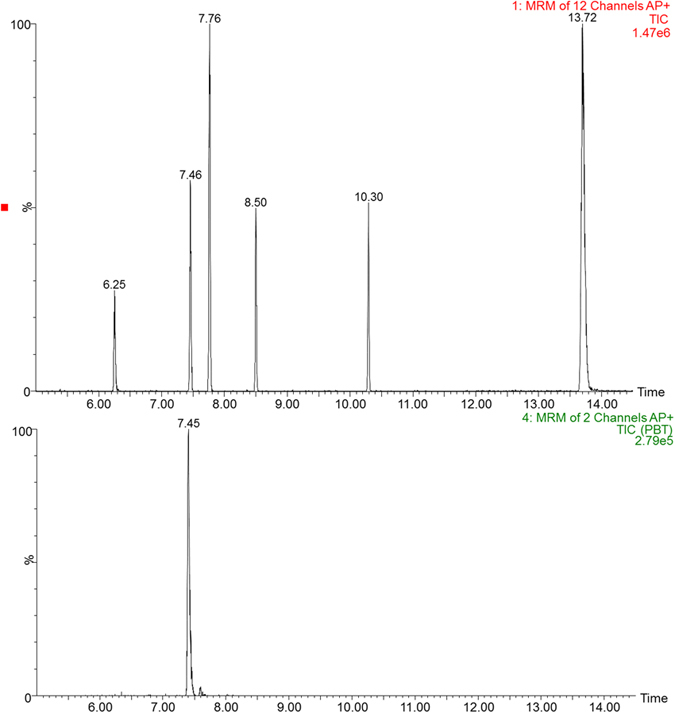
The total ion chromatogram of six eBFRs standards (top) and PBT in hairtail fish samples from Fujian province (bottom).

The pTBX and PBEB were all below the mLOQ in the analyzed samples which can be explained from relatively low detection frequencies and levels in China environment^6^,^8^. PBT, HBB and BTBPE, were the most frequently found in samples, with concentrations from <mLOQ to 351.9 pg/g w.w., <mLOQ to 39.1 pg/g w.w. and <mLOQ to 243.1 pg/g w.w., respectively. BTBPE was the most frequent detected compound (detection rate 85%) and fish was the most contaminated food. Zhou *et al*. reported BTBPE in fish samples collected from the five Great Lakes and from two lakes in Canada were all below the mLOQ (0.011 ng/g)^19^. The observations of PBT in wildlife^12^,^32^,^33^ and environment^34^, with the addition of high bioconcentration factor for aquatic species indicate that PBT might be present in foods. However, to the best of our knowledge, this is the first report that provide the direct evidence on the occurrence of PBT in foods. PBT was detected with the highest level in fish from Fujian province (351.9 pg/g w.w) (Fig. 3).

Further study is needed to trace the source of contamination in fish. However, to the best of our knowledge, this is the first report that provide the direct evidence on the occurrence of PBT in foods. DBHCTD was observed in six of twenty samples with concentration ranging from 5.5 pg/g w.w.to 101.6 pg/g w.w. All the samples from Fujian province were found with relative higher levels of PBT, HBB and BTBPE than those in samples from other three provinces. It implicates ubiquitous contamination of PBT, HBB and BTBPE in Fujian province may result from its relatively developed electrical and electronic industries. HBB was not detected in more than 200 food samples from UK and Ireland^14^ for its high LOQs. For pork sample from Sichuan province, the highest DBHCTD was observed at the level of more than 100 pg/g w.w., which suggests point source contamination.

## Conclusion

Overall, the use of APGC-MS/MS has been evaluated as an alternative approach for analyzing eBFRs in dietary samples with high sensitivities and low matrix effects. Owing to the soft ionization, the molecular ion was selected as the precursor ion and a multiple reaction monitoring (MRM) method was developed contributing to excellent selectivity and sensitivity. Moreover, the issue of peak tailing for high boiling point compound, was greatly improved by tandem capillary column technique. To the best of our knowledge, this is the first report of the occurrence of PBT, HBB, DBHCTD and BTBPE in Chinese diets. PBT, HBB and BTBPE, were ubiquitously found in Chinese dietary samples, especially with the highest concentration of PBT of 351.9 pg/g w.w. in fish sample. This application demonstrates the suitability of APGC-MS/MS for ultra trace quantification of eBFRs in dietary samples.

## Methods

### Reagents and chemicals

PBT, HBB and DBHCTD were obtained from AccuStandard Inc (NewHaven, US), pTBX, PBEB and BTBPE were purchased from Toronto Research Chemicals Inc. (Toronto, ON, Canada). Isotopically labeled standards ^13^C-HBB, ^13^C-Dechlorane (DEC) 602 and *d*_*4*_-BTBPE were purchased from Cambridge Isotope Laboratories (Andover, MA, USA) for the surrogate standards. HPLC-grade acetone, hexane and dichloromethane were supplied by Dickma (Lake Forest, CA, USA). Silica gel 60 (0.063–0.100 mm) and diatomaceous earth (EXtrelut^®^ NT) were obtained from Merck (64271 Darmstadt, Germany). Basic alumina (Al_2_O_3_), anhydrous sodium sulfate (Na_2_SO_4_) as well as concentrated sulfuric acid (H_2_SO_4_) were available from Sinopharm Chemical Reagent Co., Ltd (Beijing, China).

Prior to use, silica gel, anhydrous sodium sulfate, basic alumina and diatomaceous earth were heated at 450 °C for 12 h. Acid silica gel was prepared from activated silica gel impregnated with 44% (w/w) concentrated H_2_SO_4_ and then was shaken overnight to blend the mixture.

### Samples

Samples from 2011 Chinese TDS were used in this study. The samples included pork, chicken, egg, milk and fish collected from Sichuan, Fujian, Jilin and Henan provinces.

### Sample extraction and purification

The sample preparation for TDS has been described previously^35^.The food samples were weighed, homogenized and freeze dried. Lyophilized samples were weighed and homogenized again and stored at −20 °C until prior to analysis.

Briefly, lyophilized samples (1.0 g) were grounded with diatomaceous earth, spiked with 10 μL of 10 μg/L internal standard solutions (^13^C-HBB, ^13^C-DEC 602 and *d*_*4*_-BTBPE), and extracted with *n*-hexane/acetone (3:1, v/v) using an ASE200 accelerated solvent extractor (Dionex, USA). The ASE extraction was carried out at the temperature of 100 °C and the pressure of 1500 psi for 6 min, then 10 min in the static state, followed by 3 repeated cycles for extraction. In the end, the extraction cell was flushed with 60% of the cell volume extraction solvent and purged with nitrogen for 60 s. The extracts were collected in 100 mL bottles with Teflon septa, and then rotary evaporated to near dryness. Lipid content of the food samples was determined gravimetrically.

Sample purification was achieved by multilayer silica gel column (0.2 m × 15-mm internal diameter) which was packed with 10.0 g anhydrous sodium sulfate, 5.0 g neutral aluminum oxide, 2.0 g activated silica gel, 4.0 g acid silica gel, 2.0 g activated silica gel and 10.0 g anhydrous sodium sulfate from bottom to top and eluted with 50 ml *n*-hexane/dichloromethane (1:1, v/v). The eluent was concentrated to about 2 mL in a rotary evaporator. Then the solution was transferred to a vial to dryness under a gentle stream of N_2_ and redissolved with 100 μL *n*-hexane.

### Instruments

APGC-MS/MS. A triple quadrupole Xevo TQ-S system (Waters Corporation, Manchester, UK) equipped with APGC source operating in positive mode was used for data acquisition. This was interfaced to a modified Agilent 7890A GC system (Palo Alto, CA, USA) equipped with an Agilent 7693 auto sampler and a fused silica DB-5MS capillary column (length 15 m × I.D. 0.25 mm × film 0.10 μm) (J&W Scientific, Folson, CA, USA) for GC separation. A section of 70 cm unpacked capillary column (0.25 mm I.D., Agilent Technologies, Palo Alto, CA, USA) was connected to the end of analytical column using an ultra-inert universal press fit (0.10–0.75 mm, Agilent Technologies, Palo Alto, CA, USA). Helium was used as carrier gas in constant flow mode (1.2 mL/min). The injector was operated in 280 °C with 1 μL injection volume. A pulsed splitless injection was carried out at 60 psi for 0.9 min, and then changed to a constant flow of 1.2 mL/min. The oven temperature was programmed from an initial temperature of 100 °C, kept for 0.5 min, then increased to 250 °C at the rate of 15 °C/min and finally increased to 300 °C at 5 °C/min rate for 5 minute hold time.

The transfer line temperature was set to 350 °C. Nitrogen was used as the auxiliary gas, cone gas and make-up gas at the flow rate of 300 L/h, 150 L/h and 300 mL/min, respectively. The APCI corona discharge pin was operated at 8.0 μA. Targetlynx™ software (Waters, Manchester, UK) was used to process the acquired data.

GC-NCI-MS. GC-NCI-MS was performed on GC-MS-QP 2010 plus (Shimadzu, Kyoto, Japan) in selected ion monitoring (SIM) mode. Helium and methane were used as carrier and reagent gas, respectively. Interface and source temperature were 300 °C and 230 °C, respectively. The operating parameters of GC were same as those described for APGC-MS/MS.

GC-EI-MS/MS. GC-EI-MS/MS was performed on a Shimadzu GC-MS-TQ 8030 triple quadrupole mass spectrometer (Shimadzu, Kyoto, Japan) and the GC conditions were the same as described for APGC-MS/MS. The detector parameters used on GC-EI-MS/MS were as follows: ionization mode, EI; ionization energy, 70 eV; dwell time, 30 ms; argon gas pressure, 200 kPa; flow rate of carrier gas, 1.2 mL/min; and injection volume was set at 1 μL.

### Method Characterisation

For accurate quantification of eBFRs in sample matrices and due to similarities of analyte structure and retention times, three isotopically labeled internal standards were used: ^13^C-HBB for HBB, pTBX, PBT and PBEB, *d*_*4*_-BTBPE for BTBPE, ^13^C-DEC602 for DBHCTD.

Matrix effects were used to evaluate the clean-up procedure for all sample matrices by comparing slopes of solvent-standard calibration curves with the slopes of matrix-matched standard calibration curves. The matrix-matched standard curves were obtained by spiking standards into blank matrix extracts. The solvent-standard calibration curves and matrix-matched standards curves were depicted using absolute areas of different concentrations of eBFRs. Blank matrices were obtained by examining samples treated with the method mentioned above. If the concentration of analytes were all below method limit of detection (mLOD, signal-to-noise ratio 3), the samples were used as blank matrices.

The iLOQ and mLOQ were defined as the minimum quantified amount of analytes from standard and different food samples with signal-to-noise ratios of 10:1 in quantification transition. Recoveries of the analytes were evaluated by spiking blank pork, fish, egg, chicken and milk samples with all eBFRs at three different concentrations which was one, two and five fold of the mLOQ.

## Additional Information

**How to cite this article:** Lv, S. *et al*. Atmospheric Pressure Chemical Ionization Gas Chromatography Mass Spectrometry for the Analysis of Selected Emerging Brominated Flame Retardants in Foods. *Sci. Rep.* **7**, 43998; doi: 10.1038/srep43998 (2017).

**Publisher's note:** Springer Nature remains neutral with regard to jurisdictional claims in published maps and institutional affiliations.

## Supplementary Material

Supplementary Information

## Figures and Tables

**Table 1:** Retention time, MS conditions and MS/MS transitions of eBFRs using GC-APCI-MS/MS, GC-EI-MS/MS and GC-NCI-MS.

Name	t_R_ (min)	GC-APCI-MS/MS				GC-EI-MS/MS			GC-NCI-MS		
		Precursorion (*m/z*)	Production (*m/z*)	Loss	C.V. (V)	C.E. (eV)	Precursorion (*m/z*)	Production (*m/z*)	C.E. (eV)	Ion1 (*m/z*)	Ion2 (*m/z*)
pTBX	6.25	421.8	342.6*	-Br	25	20	422.0	342.8	20	79	81
			262.1	-Br_2_		25	341.0	102.2	40		
PBT	7.46	487.6	406.6*	-Br	25	20	488.0	406.6	20	79	81
			327.9	-Br_2_		30	326.0	246.8	15		
PBEB	7.76	501.7	486.6*	-CH_3_	25	25	500.0	484.4	25	79	81
			405.8	-CH_3_Br		35	485.0	324.7	40		
HBB	8.50	551.5	470.6*	-Br	30	35	551.0	472.3	30	79	81
			391.6	-Br_2_		40	551.0	391.6	40		
DBHCTD	10.30	539.8	142.7	-C_10_H_12_Cl_3_Br_2_	30	28	268.0	107.1	10	79	81
			105.1*	-C_10_H_12_Cl_4_Br_2_		40	107.0	79.1	10		
BTBPE	13.72	687.6	358.7*	-C_6_H_2_OBr_3_	30	25	357.0	118.1	25	79	81
			279.8	-C_6_H_2_OBr_4_		25	278.0	118.1	20		

^*^Was defined as quantitative ion.

**Table 2:** ILOQ and mLOQ for eBFRs using APGC-MS/MS, GC-NCI-MS and GC-EI-MS/MS.

	APGC-MS/MS		GC-NCI-MS		GC-EI-MS/MS	
	iLOQ (pg)	mLOQ (pg/g w.w.)	iLOQ (pg)	mLOQ (pg/g w.w.)	iLOQ (pg)	mLOQ (pg/g w.w.)
pTBX	0.05	2	0.2	15	0.5	40
PBT	0.05	2	0.2	15	0.5	40
PBEB	0.02	1	0.1	10	0.25	15
HBB	0.05	2	0.2	15	1	40
DBHCTD	0.25	8	2.5	200	5	500
BTBPE	0.05	2	0.2	25	2	100

**Table 3:** Linear range, linearity, iLOQ, mLOQ, recovery and precision of the optimized method.

eBFRs	Linear range(pg/μL)	r^2^	iLOQ (pg)	mLOQ (pg/g w.w.)	Spiked recovery of eBFRs (%)				Relative standard deviation (RSD, %) *n* = 6			
					Pork	Egg	Milk	Fish	Pork	Egg	Milk	Fish
pTBX	0.05–2	0.9951	0.05	2	83.6–96.6	81.4–95.8	89.0–96.9	82.3–109.7	<11.8	<10.0	<10.3	<10.4
PBT	0.05–5	0.9926	0.05	2	88.8–109.4	90.1–104.7	88.5–102.5	90.3–106.7	<7.7	<9.5	<8.6	<18.4
PBEB	0.02–2	0.9943	0.02	1	88.2–100.3	83.3–111.1	92.3–111.8	88.9–119.6	<14.2	<12.6	<13.7	<12.7
HBB	0.05–2	0.9959	0.05	2	94.4–104.5	90.0–101.9	94.0–106.2	84.7–111.9	<8.2	<7.6	<9.9	<16.7
DBHCTD	0.25–5	0.9926	0.25	8	81.1–118.3	81.6–109.9	81.2–119.5	84.2–101.5	<18.6	<19.6	<17.9	<10.0
BTBPE	0.05–2	0.9948	0.05	2	87.4–111.5	80.4–115.1	99.7–119.3	81.1–89.2	<13.3	<17.7	<12.7	<13.6

**Table 4:** The results of eBFRs concentrations in dietary samples (pg/g w.w.).

Sampling Location	Sample	pTBX	PBT	PBEB	HBB	DBHCTD	BTBPE
Sichuan	Pork	nd	31.2	nd	7.1	101.6	4.9
	Chicken	nd	29.9	nd	nd	nd	80.9
	Egg	nd	nd	nd	nd	nd	nd
	Milk	nd	7.5	nd	5.3	nd	10.0
	Fish	nd	6.7	nd	4.5	nd	70.2
Fujian	Pork	nd	40.6	nd	5.7	nd	19.5
	Chicken	nd	48.7	nd	4.3	nd	69.1
	Egg	nd	20.7	nd	nd	nd	141.6
	Milk	nd	3.5	nd	6.6	nd	nd
	Fish	nd	351.9	nd	39.0	39.0	243.1
Jilin	Pork	nd	nd	nd	nd	nd	3.1
	Chicken	nd	7.2	nd	2.8	17.5	20.1
	Egg	nd	2.8	nd	3.9	nd	17.6
	Milk	nd	3.1	nd	2.1	14.8	nd
	Fish	nd	nd	nd	nd	nd	32.2
Henan	Pork	nd	nd	nd	nd	nd	10.4
	Chicken	nd	nd	nd	nd	12.6	80.1
	Egg	nd	nd	nd	nd	nd	38.2
	Milk	nd	2.1	nd	nd	nd	5.9
	Fish	nd	nd	nd	nd	nd	52.5

nd: not detected or below the mLOQ.
