# Supplementary material for: Atmospheric Pressure Chemical Ionization Gas Chromatography Mass Spectrometry for the Analysis of Selected Emerging Brominated Flame Retardants in Foods
Source: Sci Rep. 2017 Mar 10;7:43998. doi: 10.1038/srep43998 (PMC5345032; doi:10.1038/srep43998)
Supplement: Supplementary Information [file srep43998-s1.pdf]

Supporting Information

Atmospheric Pressure Chemical Ionization Gas Chromatography Mass Spectrometry for the Analysis of Selected Emerging Brominated Flame Retardants in Foods

Surong Lv[ζ†], Yumin Niu[‡],Jing Zhang[‡], Bing Shao[ζ*],Zhenxia Du[†*]
[ζ]Beijing Advanced Innovation Center for Food Nutrition and Human Health,China Agricultural University, Beijing 100193, China
[†]College of Science, Beijing University of Chemical Technology, Beijing, 100029,China
[‡]Beijing Key Laboratory of Diagnostic and Traceability Technologies for Food poisoning, Beijing Center for Disease Prevention and Control, 100013, China

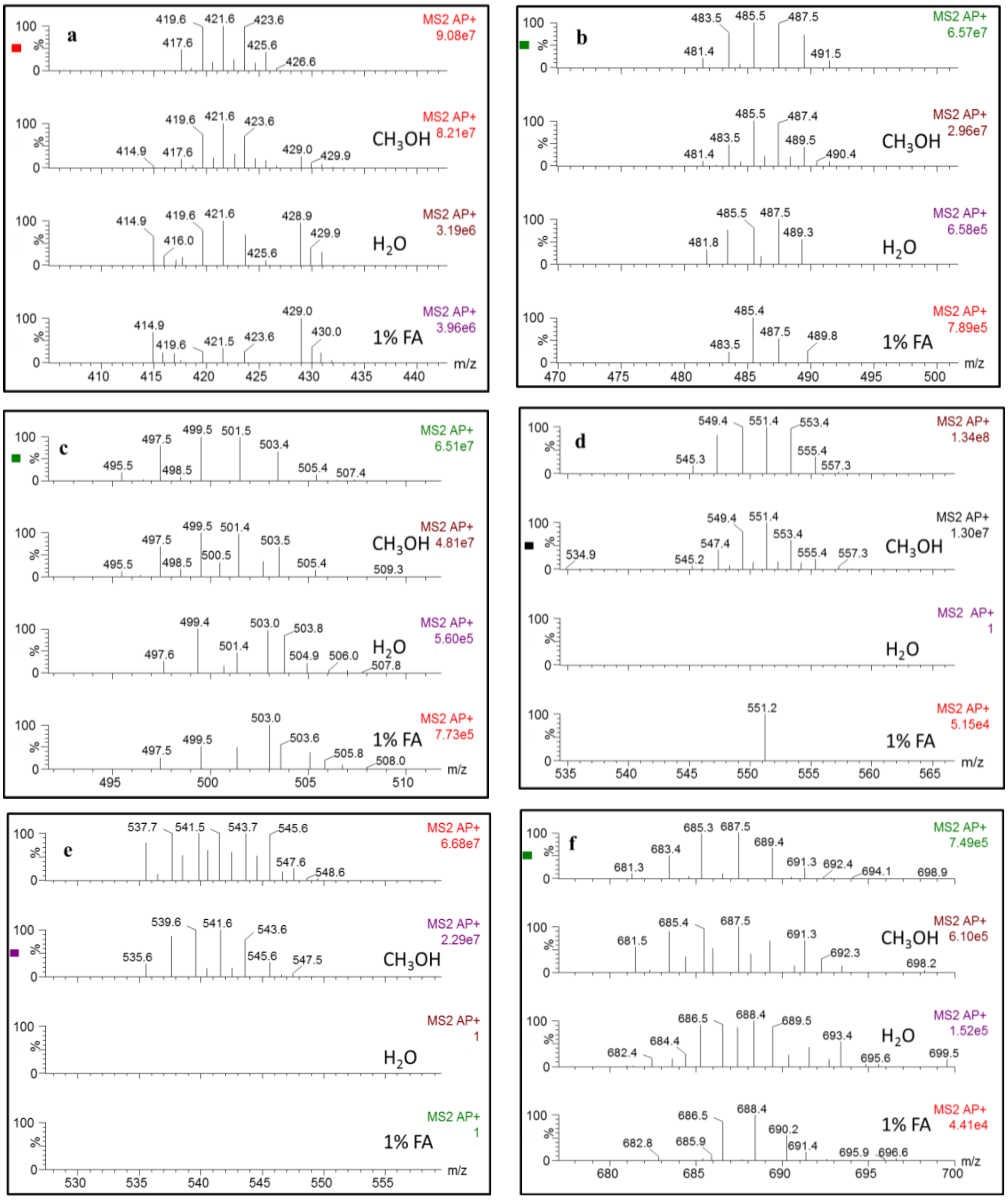

Supplementary Figure S1.The APGC-MS spectrums of six eBFRs solution (a-pTBX; b-PBT; c-PBEB; d-HBB; e-DBHCTD; f-BTBPE) obtained in full scan positive mode using different modifiers (methanol-CH_3OH; water-H_2O; 1% formic acid water solution-1% FA).

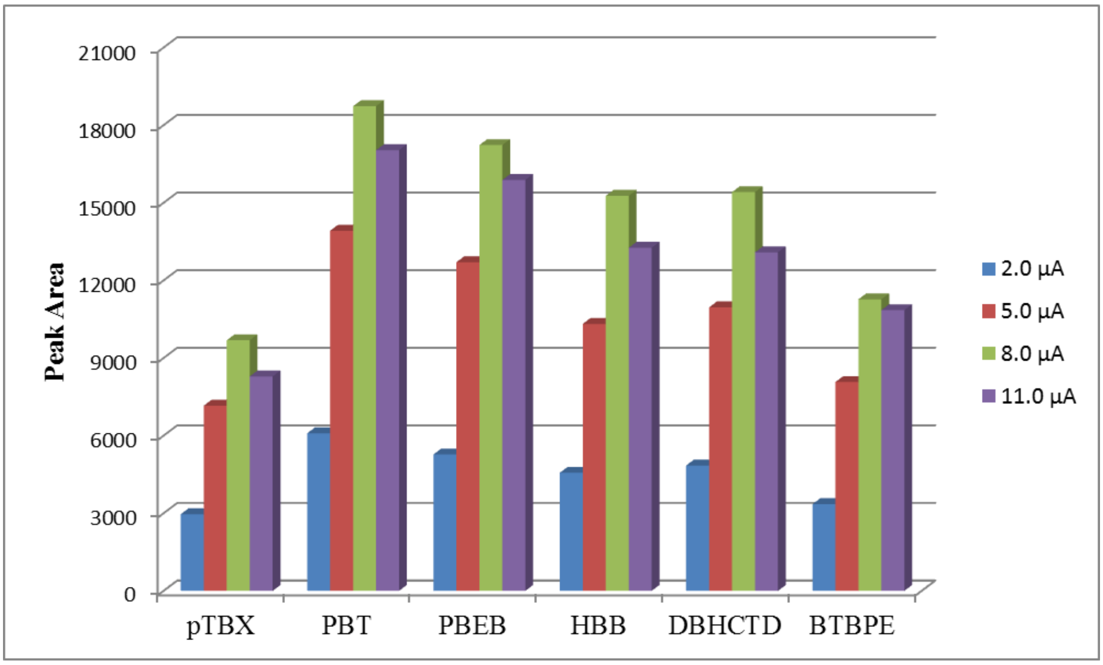

Supplementary Figure S2. The response of eBFRs at corona current values of 2.0 μA, 5.0 μA, 8.0 μA and 11.0 μA.

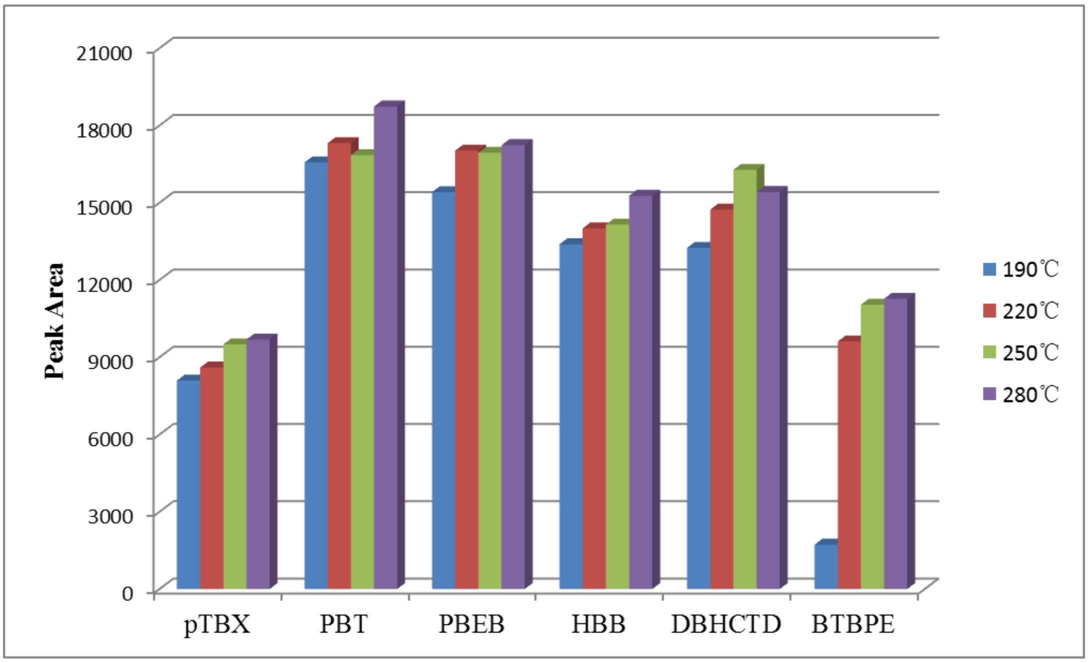

Supplementary Figure S3. The response of eBFRs at injector temperature of 190°C, 220°C, 250°C and 280°C.

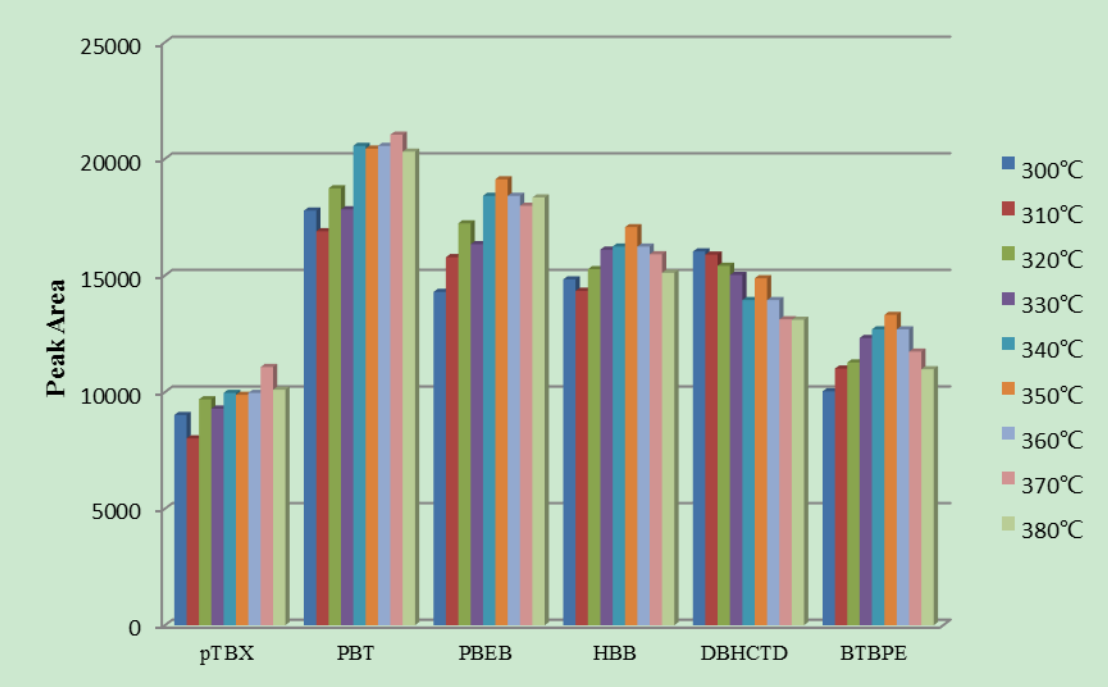

Supplementary Figure S4.The response of eBFRs at transfer line temperature between 300°C to 380°C with an interval of 10°C.

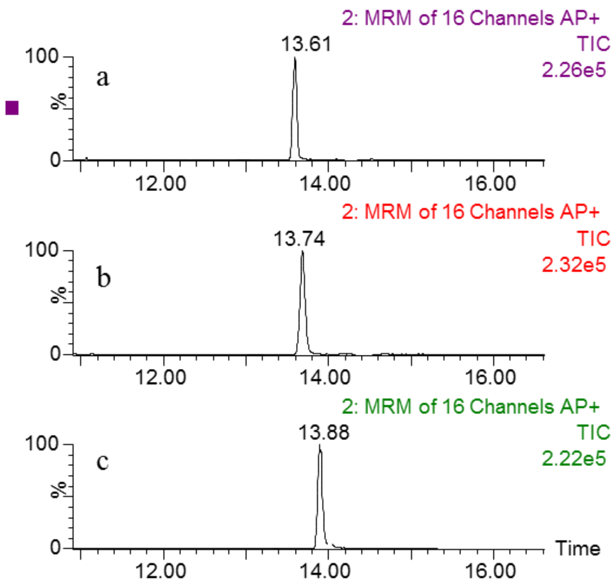

Supplementary Figure S5. The chromatographams of BTBPE with the length of unpacked column at 55 cm (a), 70 cm (b) and 85 cm (c)

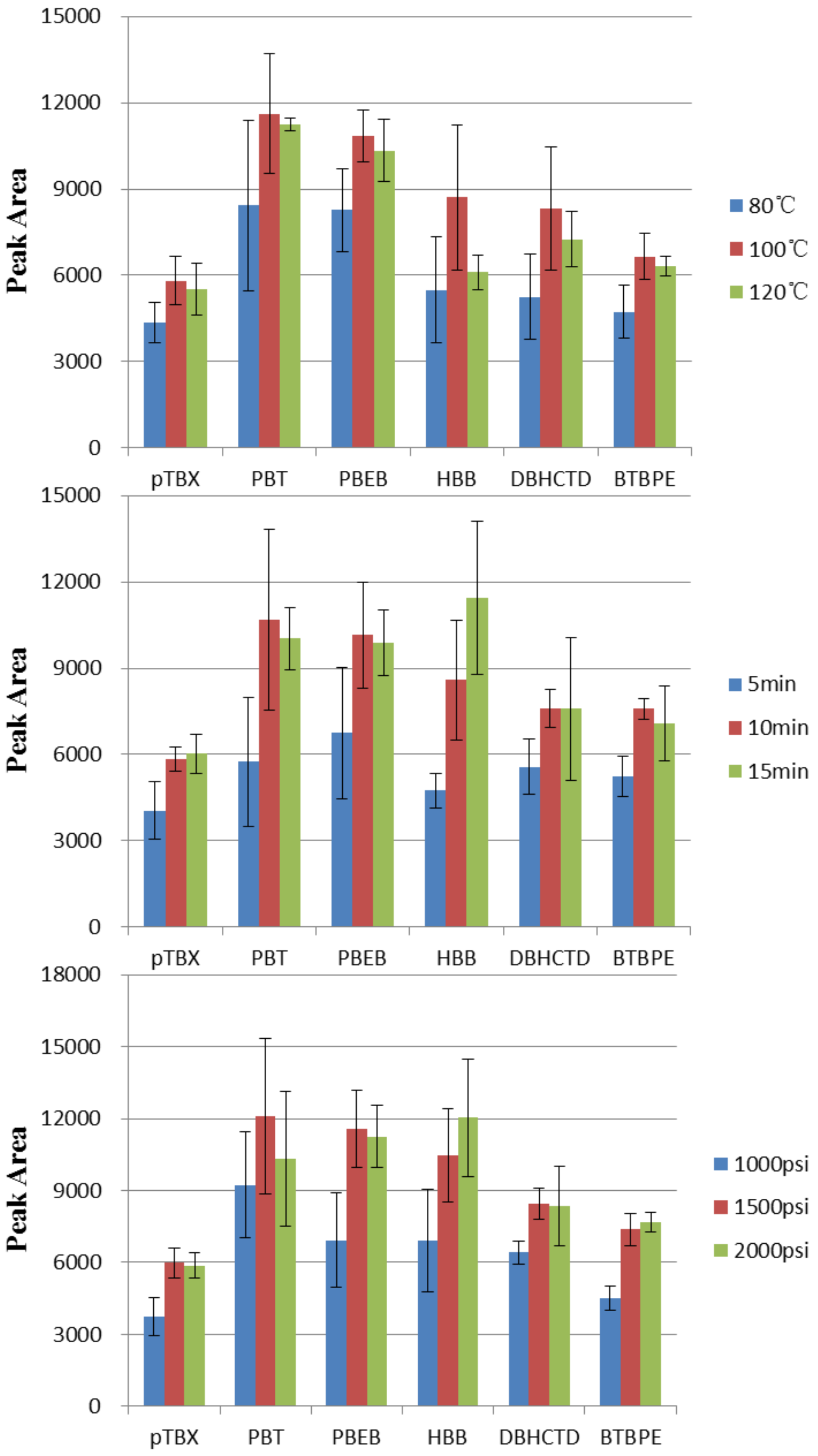

Supplementary Figure S6. The influence of extraction temperature, time and pressure for recovery on the extraction of eBFRs using accelerate solvent extraction.

Supplementary Table S1. The information of targeted eBFRs in this study.

Name	CAS No.	Formula	MW	Chemical Structure
pTBX	23488-38-2	$C_8H_6Br_4$	421.75	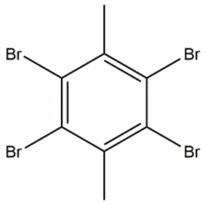
PBT	87-83-2	$C_7H_3Br_5$	486.62	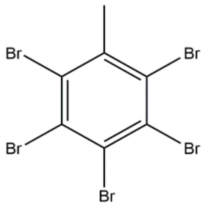
PBEB	85-22-3	$C_8H_5Br_5$	500.64	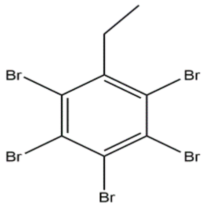
HBB	87-82-1	C_6Br_6	551.48	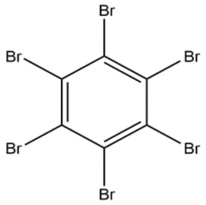
DBHCTD	51936-55-1	$C_{13}H_{12}Br_2Cl_6$	540.76	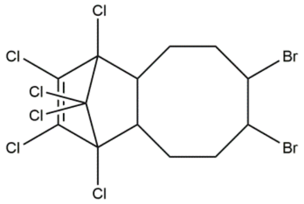
BTBPE	37853-59-1	$C_{14}H_8Br_6O_2$	687.64	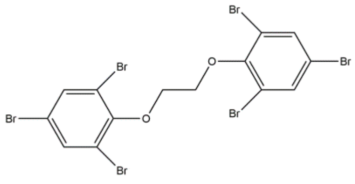

Supplementary Table S2. The matrix effects of analytes in fish, chicken, egg and milk matrix.

	pTBX	PBT	PBEB	HBB	DBHCTD	BTBPE	^{13}C-HBB	^{13}C-DEC602	d4-BTBPE
Fish	0.73	0.95	0.90	0.72	0.96	1.09	0.74	0.81	1.06
Chicken	0.85	0.90	0.92	0.91	0.79	0.98	0.83	0.80	1.10
Egg	0.83	0.96	0.88	0.82	0.74	0.86	0.89	0.92	0.85
Milk	0.81	0.75	0.88	0.82	0.74	0.85	0.77	0.86	0.86

Matrix effects were evaluated by comparing slopes of solvent-standard calibration

curves with the slopes of matrix-matched standard calibration curves.
